# Antioxidant and Protective Mechanisms against Hypoxia and Hypoglycaemia in Cortical Neurons *in Vitro*

**DOI:** 10.3390/ijms15022475

**Published:** 2014-02-12

**Authors:** José Joaquín Merino, César Roncero, María Jesús Oset-Gasque, Ahmad Naddaf, María Pilar González

**Affiliations:** 1 Departamento de Bioquímica y Biología Molecular II, Facultad de Farmacia, Universidad Complutense de Madrid (U.C.M.) Ciudad Universitaria, Madrid 28040, Spain; E-Mails: ceronce@ucm.es (C.R.); mjoset@ucm.es (M.J.O.-G.); 2 Faculty of Pharmacy, Isra University, PO Box 22, Amman 11622, Jordan; E-Mail: drahmadnaddaf@hotmail.com

**Keywords:** cortical neurons, cerebral ischemia, ROS formation, antioxidant defenses, mitochondrial membrane potential, free radical, SOD1/caspase-3

## Abstract

In the present work, we have studied whether cell death could be induced in cortical neurons from rats subjected to different period of O_2_ deprivation and low glucose (ODLG). This “*in vitro*” model is designed to emulate the penumbra area under ischemia. In these conditions, cortical neurons displayed loss of mitochondrial respiratory ability however, nor necrosis neither apoptosis occurred despite ROS production. The absence of cellular death could be a consequence of increased antioxidant responses such as superoxide dismutase-1 (SOD1) and GPX3. In addition, the levels of reduced glutathione were augmented and HIF-1/3α overexpressed. After long periods of ODLG (12–24 h) cortical neurons showed cellular and mitochondrial membrane alterations and did not recuperate cellular viability during reperfusion. This could mean that therapies directed toward prevention of cellular and mitochondrial membrane imbalance or cell death through mechanisms other than necrosis or apoptosis, like authophagy, may be a way to prevent ODLG damage.

## 1. Introduction

The brain requires a continuous supply of oxygen and glucose to maintain central nervous system (CNS) functions. The deprivation resulting from stroke or respiratory failure, can rapidly lead to transient or permanent neuronal injury as consequence of reduced energy bioavailability; pump function or membrane integrity [1]. Cerebral ischemia is an important cause of death and working disability in humans [2,3]. Caspase-3, an effecter enzyme involved in apoptosis [4], may contribute to the pathophysiology of cerebral ischemia. Other mechanisms including inflammation and angiogenesis together with ROS formation contribute to cell death in stroke [5–8]. However, ROS production protects against H_2_O_2_ toxicity [9] and may regulate signal transduction pathways under physiological [10,11] and pathological conditions [12–15]. However, controversial data suggests that glutamate may play a neurotoxic role in cerebral ischemia [16–23].

Cerebral artery occlusion can induce apoptosis or necrotic cell death. This occlusion may induce differential damages depending of the lack of O_2_ and nutrients. These zones are the “core” area and the “penumbra” area, which are rendered functionally silent but retain alive cells [24]. Many studies have shown that neurons from the ischemic penumbra undergo apoptosis rather than necrosis, suggesting that this area can be potentially and functionality recovered [25–27], however, the molecular mechanisms involved are not well known.

Hypoxia-inducible factors (HIFs) are transcriptional mediators of adaptive responses that decrease oxygen availability, a characteristic of ischemic-hypoxia conditions [28]. HIFs are ubiquitously expressed in several mammalian cell types [29]. Since its discovery, the HIFs cascade has been extensively studied and plays a role in both physiological, as tissue repair, and pathological process [30–32], as ischemia [33,34].

The model of oxygen deprivation-low glucose (ODLG) chosen for our study is a reliable and validated method to study cellular damage in cortical neurons subjected to partial ischemia (penumbra area). In our study, cultured cortical neurons were subjected to oxygen-deprivation and low glucose (ODLG model). We studied whether neuronal damage, at different ODLG times (1, 3, 5, 12, and 24 h) could exist under these vulnerability conditions, by evaluating whether (1) ROS formation and expression of antioxidant enzymes (SOD-1 and GPX3) together with the mitochondrial enzyme cytochrome oxidase (CO) might be differentially regulated under ODLG; (2) to study whether HIF-1/3α stabilization could prevent neuronal loss in these cortical neurons; (3) evaluate whether cellular and membrane mitochondrial integrity were affected by measuring membrane potential after ODLG as well as (4) possible cellular dysfunctions of neurotransmission during reperfusion by testing Asp, Glu, Gly and GABA release by HPLC.

## 2. Results

### 2.1. Presence of “HIF” Factor during Hypoxia

**Figure 1.**
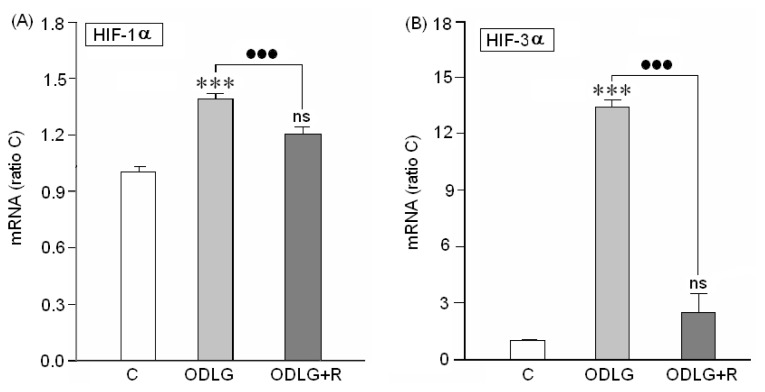
Induction of HIF 1α (**A**) and HIF-3α gene expression (**B**) in cortical neurons after exposure to ODLG conditions during 24 h. C = control, ODLG = 24 h treatment, ODLG + R = 24 h treatment followed by 24 h reperfusion. ns = no significant (*vs.* control) (***) or (●●●) = *p* < 0.005; (***) = *vs.* control; (●●●) = *vs.* ischemia with or without reperfusion.

The first objective was to demonstrate whether the cortical neurons cultured, under hypoxia, are truly under anoxic conditions. It has been reported that transcription factors (HIF) can be induced in cellular and animal models of cerebral ischemia [35,36]; consequently, the presence of this factor in our preparations was checked by RT-PCR. Data in Figure 1 indicate that under our experimental conditions, cortical neurons significantly upregulated HIF-1α and HIF-3α transcription factors. Reperfusion reversed HIF-1α and HIF-3α upregulation although the values were always higher than the controls.

### 2.2. ROS Formation

**Figure 2.**
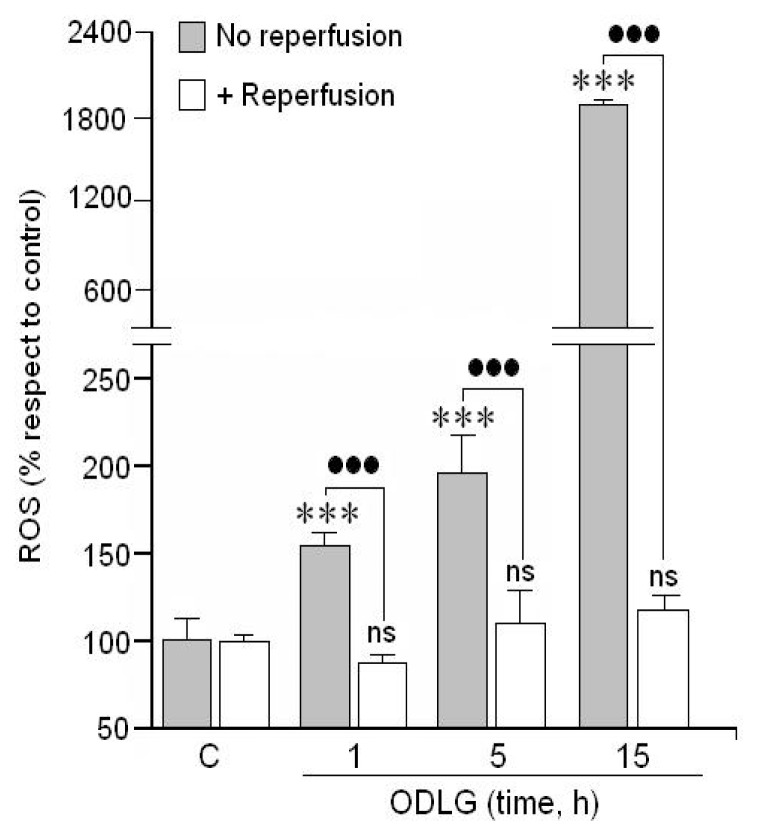
Effect of different ODLG treatment time on ROS formation. Reperfusion = Cortical neurons after the ODLG treatments were subjected to 24 h under a normal atmosphere after the ODLG treatment. Results are means ± SEM of three experiments with cells from different cultures, each one performed in triplicate with different batches of cells (9 measurements/condition). ns = no significant (*vs.* control); (***) or (●●●) = *p* < 0.005, (*) (*vs.* control), (●) (*vs.* ischemia with or without reperfusion).

Since oxygen radicals (ROS) are suspected to be involved in cellular injury and death, we analyzed whether hypoxia and hypoglycemia (ODLG) could modulate ROS production “*in vitro*”. Results in Figure 2 indicate that cortical neurons subjected to these hypoxic and hypoglycemic conditions, generated ROS at the moment in which the cells were in a normal atmosphere. This ROS production increased progressively under hypoxia. In addition, when these neurons were subjected to 24 h of reperfusion, ROS production was completely reversed, reaching values similar to those in control cells.

### 2.3. Cell Viability

**Figure 3.**
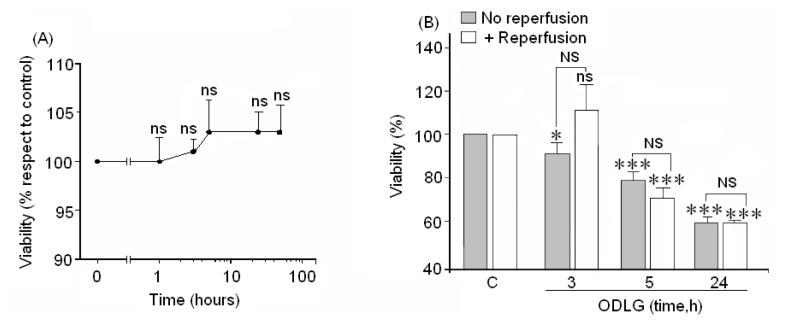
(**A**) Cells were subjected to ODLG for 0 to 48 h before evaluating cell viability with the crystal violet method; (**B**) Cells were subjected to 3, 5 or 24 h of ODLG times, followed or not by reperfusion. Then, cell viability was measured by the XTT assay. Results are expressed as means ± SEM from two/three experiments with cells from different cultures, each experiment performed in triplicate with different batches of cells (6–9 measurements/condition). ns = no significant (*vs.* control); NS = no significant (*vs.* ischemia with or without reperfusion); (*) = *p* < 0.05 (*vs.* control); (***) = *p* < 0.005 (*vs.* control).

Since ROS formation could play a pivotal role in cell death, cell viability was tested by two methods, crystal violet, which measures cell viability (Figure 3A) and the XTT assay, which measures the metabolic activity of these neurons (Figure 3B). The crystal violet test showed an absence of neuronal loss in cortical neurons at all studied times (Figure 3A). However, the XTT assay showed a time-dependent loss of cell viability (Figure 3B). Reperfusion did not reverse the loss of viability in cells subjected to 5 to 24 h of ODLG, although at shorter times showed levels similar to controls.

### 2.4. Cellular Death (LDH Assay)

**Figure 4.**
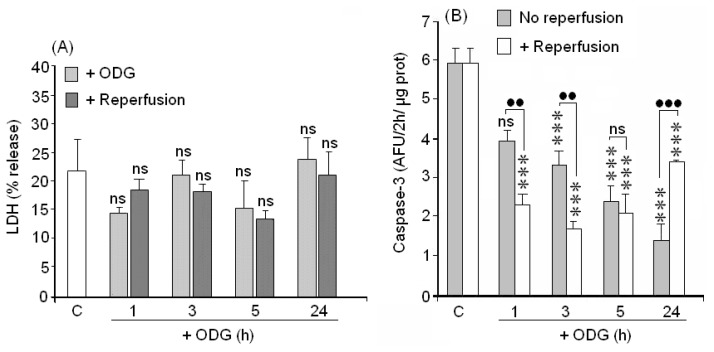
Evaluation of cellular death. (**A**) Necrosis was evaluated by LDH release; (**B**) Apoptosis was evaluated by caspase-3 activity. Results are means ± SEM of three experiments with cells from different cultures, each one was performed in triplicate with different batches of cells (9 measurements/condition). ns = no significant (*vs.* control); (●●) = *p* < 0.01 (*vs*. ischemia with or without reperfusion); (***) (*vs*. control) or (●●●) = *p* < 0.005 (*vs*. ischemia with or without reperfusion).

The lack of cellular viability in the XTT assay has two interpretations: (1) cell death or (2) loss of metabolic ability. In order to check these options, LDH activity was also measured to evaluate necrotic death and caspase-3 activation (a sensor of apoptosis) was quantified. Results in Figure 4A show that cortical neurons at 1 to 24 h of ODLG did not significantly increase their LDH release.

### 2.5. Caspase-3 Activation

Regarding caspase-3 activity, there was a significant proportional decline in caspase-3 activity with the increase in ODLG times (Figure 4B). Reperfusion did not reverse the caspase-3 inactivation produced by 1–5 h of ODLG. In fact, caspase 3 activity was even lower during reperfusion at this time. The inhibitory effects were more evident after 1–3 h of ischemia. Caspase-3 was significantly overproduced at 24 h during reperfusion as compared to values obtained in the non reperfused cells.

### 2.6. Cytochrome Oxidase

**Figure 5.**
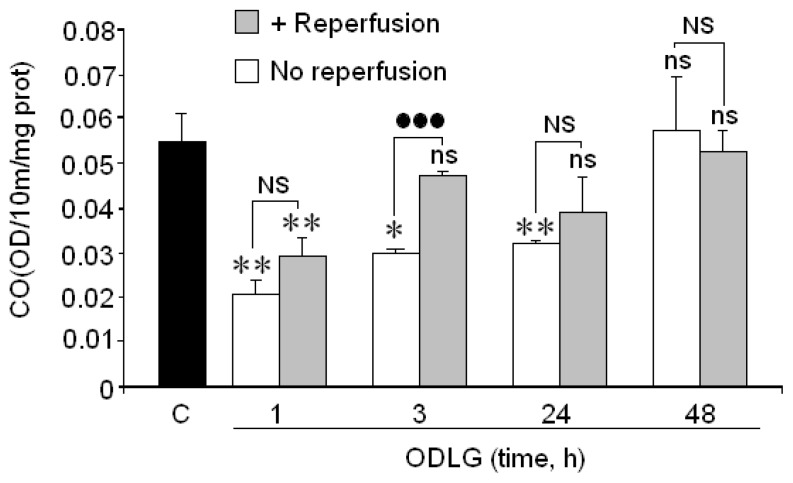
Effect of 1 to 48 h of ODLG on *cytochrome oxidase* activity. Results are means ± SEM in two experiments with cells from different cultures, each one performed in triplicate with different batches of cells (6 measurements/condition). ns = not significant (*vs.* control); NS = no significant (*vs.* ischemia with or without reperfusion); (*) = *p* < 0.05 (*vs.* control); (**) = *p* <0.01 (*vs.* control); (●●●) = *p* < 0.005 (*vs.* ischemia with or without reperfusion).

Results in Figure 5 show that cytochrome oxidase activity (CO), the terminal enzyme of the respiratory chain, decreased during the first hour of ODLG but returned to basal values at longer hypoxia times. During reperfusion, CO decreased at 1 h of ODLG and reached values close to control levels at 3 to 48 h of ODLG.

### 2.7. Possible Mechanisms which Protect against ROS Damage

**Figure 6.**
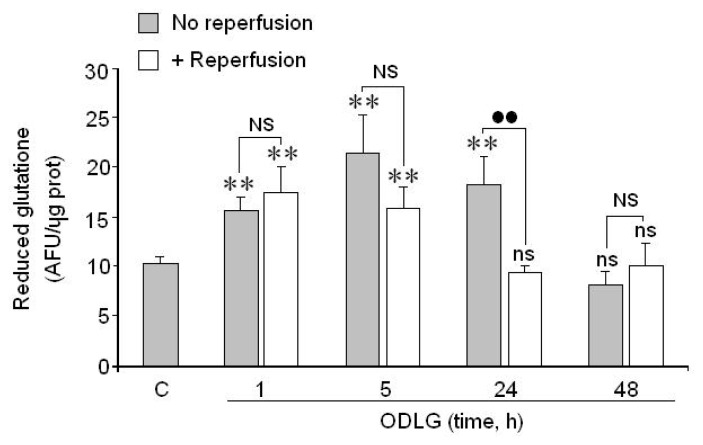
Action of 1, 3, 5, 24 and 48 h of ODLG treatment on reduced glutathione levels, before and after 24 h of reperfusion. Results are means ± SEM of two experiments with cells from different cultures, each one performed in triplicate (6 measurements/condition). ns = no significant (*vs*. control); NS = no significant (*vs*. ischemia with or without reperfusion; (**) (*vs*.control) or (●●) (*vs*. ischemia with or without reperfusion) = *p* < 0.01.

**Figure 7.**
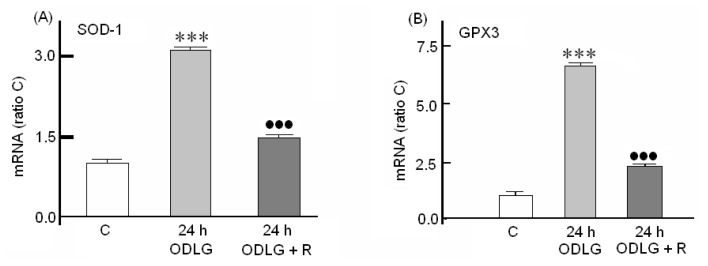
Effect of ODLG treatment on (**A**) SOD1 gene and (**B**) on GPX3 gene induction. ODLG = 24 h of ODLG exposure, ODLG + R = 24 h of ODLG exposure plus 24 h reperfusion. (***) (*vs.* control) or (●●●) (ODLG *vs*. ODLG + R) = *p* < 0.005.

As our results do not seem to indicate the existence of cellular death, even despite the high ROS formation, it seems possible that cortical neurons were able to activate defense mechanisms against ROS levels. In fact, glutathione (Figure 6), SOD1 and GPX3 (Figure 7A,B) levels were significantly increased during the first 24 h of ODLG treatment. At 48 h of ODLG, glutathione reached values similar to controls. Reperfusion reversed the SOD1 and GPX3 upregulation although these values still remained higher than in controls (Figure 7A,B). Conversely, during reperfusion, when cells were subjected to 1 to 5 h of hypoxia, glutathione levels were higher than in controls. However, these values were similar to controls at 24 and 48 h of ischemia (Figure 6).

### 2.8. Cellular and Mitochondrial Membrane Potential

**Figure 8.**
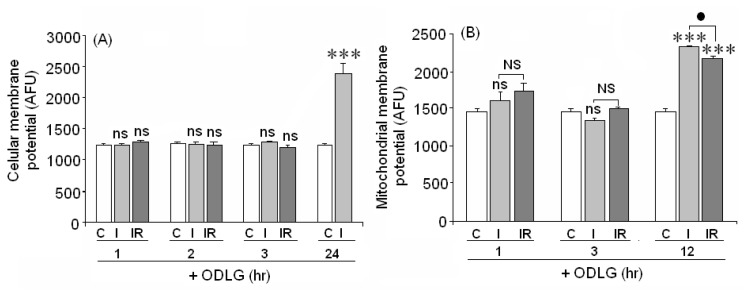
(**A**) Effect of 1, 2, 3 and 24 h of ODLG exposure on cell membrane potential; (**B**) Effect of ODLG on cell mitochondrial membrane potential at different ODLG exposure time. Results are means ± SEM of two experiments with cells from different cultures, each one performed in triplicate with different batches of cells (6 measurements/condition). ns = not significant (*vs.* control); NS = no significant (*vs.* I and IR); (●)= *p* < 0.05 (*vs.* I and IR) and (***)= *p* < 0.005 (*vs.* control).

Twenty-four h of hypoxia plus hypoglycemia enhanced cellular membrane potential, suggesting that membrane depolarization was induced by ODLG (Figure 8A). However, low levels of ODLG did not alter the cellular membrane potential. A similar effect was observed in the mitochondrial membrane potential (PMM), which was increased at 12 h of ODLG (Figure 8B), again suggesting depolarization. These effects were not reversed during reperfusion. Shorter ODLG treatment times (1–3 h) did not affect the mitochondrial membrane potential.

### 2.9. Cellular Functionality (Amino Acid Release)

**Figure 9.**
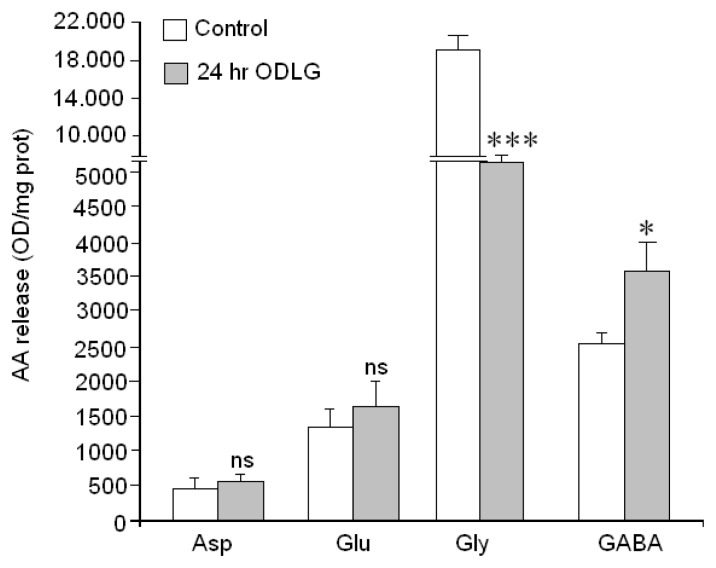
Effect of ODLG on amino acid neurotransmitter release evoked by high potassium levels (60 mM). Results are means ± SEM of two experiments with cells from different cultures, each one performed in triplicate with different batches of cells (6 measurements/condition). The statistical significance was assessed by comparisons between controls and cells subjected to ODLG for 24 h. ns = not significant (*vs*. control); (*) = *p* < 0.05 (*vs*. control); (***) = *p* <0.005 (*vs*. control).

Finally, since neurotransmission regulates neuronal function, we used HPLC to measure amino acid neurotransmitter release mediated by high KCl in neurons subjected to ODLG for 24 h as well as in controls neurons (Figure 9). Our data indicate that Gly and GABA release were affected as compared to control release in controls. Gly release was higher in control than in ODLG-treated neurons; however the high KCl-induced GABA release was higher in treated neurons as compared to their controls. Nevertheless, ODLG treatment did not affect Asp and Glu release, suggesting that ischemia had only affected inhibitory synapses “*in vitro*”.

## 3. Discussion

### 3.1. HIF1α and HIF3α Are Upregulated during ODLG

Our data clearly demonstrate that, under ODLG hypoxia factors HIF1α and HIF3α are upregulated in cortical neurons “*in vitro*”. This suggests that cortical neurons could develop adaptative responses to O_2_ deprivation “*in vitro*”. Although HIF1α levels were lower during reperfusion, they were still higher than in control conditions. In concordance with our findings, several authors have reported that HIF 1α and HIF 3α upregulation can prevent cell death from hypoxia [37,38]. Wu *et al*. [39] point out that HIF-1α contributes to early brain injury (EBI) after subarachnoid hemorrhage consisting of cell apoptosis, blood-brain-barrier (BBB) disruption, and brain edema through VEGF upregulation. These detrimental effects were prevented in the presence of the VEGF inhibitor, 2ME2. Our findings suggest that HIF-1α and HIF-3α upregulation may lead to adaptative responses against O_2_ deprivation in cortical neurons during hypoxia and even during reperfusion because although during this time the levels of HIF-1α and HIF-3α were decreased, the levels of these molecules were slightly higher than in controls.

### 3.2. ROS Generation and Antioxidant Mechanisms

Unexpectedly, cortical neurons were able to survive in our experimental conditions of ODLG despite the very high ROS production. Thus, we are interested to understand the mechanisms involved in cell protection during this time. Our data indicate that increased glutathione together with SOD1 and GPX3 overproduction under ODLG and even during reperfusion may prevent ROS-mediated damage in cortical neurons “*in vitro*”. In support of the antioxidant effects observed in our study, Chen *et al*. [40] showed that SOD1 was increased during the ischemia in astrocytes culture following oxygen glucose deprivation and reperfusion.

Thus, SOD-1 overexpression may be an essential antioxidant mechanism that prevents neuronal death. Another study in rabbit spinal cords agreed with our findings and indicated that SOD1 protected neuron from spinal ischemic damage by both decreasing lipid peroxidation and maintaining catalase levels [41].

Collectively, these observations suggest that SOD-1 together with GPX3 overexpression is an adaptative response to oxygen deprivation that also occurred in our study. The reduced antioxidant SOD1 and GPX3 levels observed during reperfusion could decrease antioxidant responses during this time; this would coincide with strong caspase-3 activation during the reperfusion of cortical neurons subjected to ODLG during 24 h in the present study.

Another point deserving mention is the formation of ROS during hypoxia/reperfusion. ROS formation requires O_2_ and, under O_2_ deprivation (hypoxia), cortical neurons are in an atmosphere of 95% N_2_/5% CO_2_. ROS formation during hypoxia has been reported by several authors, who demonstrated ROS generation in complex III of the mitochondria respiratory chain during hypoxia [42]. In our opinion, the presence of ROS during hypoxia would be possible because during this process, NADH may accumulate. At the moment that cells are rescued from hypoxia, they have enough O_2_ to produce ROS despite the recent hypoxic conditions. Thus, ROS would not be formed during hypoxia although NADH might further accumulate. In fact, Livnat *et al*. [43] found increased NADH levels in the core and penumbra areas.

### 3.3. Cytochrome Oxidase and ODLG

During ODLG, cytochrome oxidase is lower during the first hour of hypoxia in cortical neurons but afterwards the enzyme shows a progressive recuperation in which it can even reach values similar to the control. This rise could reflect adaptative responses under vulnerability conditions. Cellular adaptation during ischemia has been previously reported by others who showed that preconditioning can protect against subsequent cellular insults [44–46].

### 3.4. ODLG and Cellular Death

Although neuronal death by necrosis or apoptosis did not occur under ODLG conditions, there were deficits in physiological cellular respiration together with defective neurotransmission that could affect normal neuronal function. The decreases in metabolic ability generated during reperfusion could be due to ATP depletion. If ATP levels were depleted, caspase-3 might not be activated during reperfusion (as occurred in our experiments). As a consequence, neuronal survival could be preserved during this period such as has been reported by Mattson and coworkers [47]. ATP depletion in cortical neurons during oxygen glucose deprivation (OGD) has been reported by others [48,49]. The lack of ATP does not necessarily induce cell death; thus, Yager *et al*. [50], studying hypoxic-ischemic damage in astrocytes, found that low ATP was not responsible for cell death. On the other hand, the lethargic state of cortical neurons during ODLG observed in our study by the XTT assay may protect them since such mechanisms can save energy, nutrients and O_2_ under vulnerable conditions. However, the lack of neuronal viability during reperfusion after 5 and 24 h of ODLG in our study suggests that: (1) there was cellular death or (2) there was an absence of recovery of the normal status during reperfusion. Through the crystal violet test or LDH and caspase-3 assays, our results indicated the absence of cell death by necrosis or apoptosis after 5 to 24 h of ODLG. Thus, we should not exclude the possibility that autophagy could have occurred during this time period. In fact, Arce *et al*. [50] showed that cortical neurons subjected to oxygen-glucose-deprivation (OGD) regulated BCL3, a protein involved in autophagy. Moreover, Zhang *et al*. [51] have reported that cerebral ischemia-reperfusion induces autophagy as a protective mechanism against neuronal injury.

### 3.5. ODLG and Cellular Malfunction

Additionally, cortical neurons may malfunction after a long period of ODLG since defective neurotransmission was detected together with dysfunctions in cellular and mitochondrial membrane potentials. Reperfusion after ischemia is a critical period that can lead to cell death as a consequence of mitochondrial membrane alterations during ODLG. In our study, the mitochondrial membrane potential increased at 12 h of ischemia, indicating membrane depolarization. However, this increase remained high after 24 h of reperfusion. These findings suggest that mitochondrial alterations cannot be reversed by reperfusion after a long ODLG treatment, so, the use of membrane protectors could preserve cellular integrity during this period. Our results suggest that mitochondrial alterations after ODLG may contribute to neuronal vulnerability even though apoptosis was not observed in our study. On the other hand, our results also show cellular membrane depolarization after 24 h of ODLG. This could affect neurotransmitter release (mainly glutamate) [47,48] under anoxia and hypoglycemia. Nevertheless, glutamate does not seem to be responsible for cellular death in our study, given the absence of cell death that was found in our experimental conditions.

As a whole, our findings suggest that ODLG predisposes cortical neurons to cellular vulnerability and could resemble some neurotoxic mechanisms within the penumbra of ischemic rats and humans. In these conditions, cells are able to survive by inducing compensatory mechanisms that reduce neurodegeneration. The relevance of our findings is its potential to prevent cell death and repair damaged neurons in cerebral ischemia. The lack of O_2_ and low glucose could predispose neurons to vulnerable conditions as in the penumbral area, through ROS production. Nonetheless, cells are capable of surviving through compensatory defense/protective mechanisms under these vulnerable conditions (antioxidant defenses). However, part of these mechanisms could be lost at reperfusion, mainly as a consequence of mitochondrial membrane alteration under hypoxia. Cells under hypoxia could activate endogenous antioxidant mechanisms to ameliorate cell death and compensate for mitochondrial membrane imbalances [50]. Thus, the use of antioxidants does not seem to protect neurons during reperfusion because there was a reversion of ROS formation during this period. During induced ODLG, neurons are able to activate endogenous antioxidant responses. Thus, the use of cellular and mitochondrial membrane protectors may be a good way to protect neurons during reperfusion after ischemia.

## 4. Experimental Section

### 4.1. Cell Isolation and Culture

Brains from fetal (E19) rats of 19 days gestation were used. Brain neurons were obtained according to the procedure described by Segal [35] with minor modifications. Isolated neurons were suspended in EMEM medium with addition of 0.3 g/L glutamine, 3 g/L glucose, 10% FCS, 100 U/mL penicillin and 100 μg/mL streptomycin. Cells, at a density of 10^6^ cells/cm well, were placed on plastic Petri dishes treated with 10 μg/mL of poly-d-lysine, to attach them to the plates. Cells were then incubated in a humidified incubator with 5% CO_2_/95% air at 37 °C. After 72 h, the incubation medium was replaced with a fresh medium containing 10 μM of cytosine arabinoside to prevent glial overgrowth in the culture medium. Cells were studied after seven days “*in vitro*” (7 DIV). Cell purity was checked by staining cells both with cresyl violet to identify neurons and anti-GFAP antibody to identify atrocytes.

Glial contamination was tested following the procedure from Figueroa *et al*. [36].

### 4.2. ODLG Treatment

All experiments were performed at seven days of culture. The cultured medium was removed and replaced by an EMEM commercial medium containing 1 g/L glucose and 10% of fetal calf serum (low glucose). This treatment was performed on two sets of cells. Cells were placed for indicated time, in a chamber at 37 °C within an atmosphere of 95% N_2_/5% CO_2_ (O_2_ deprivation). After different times of ODLG, a set of cells was taken and several biochemical markers were assayed.

Parallel control experiments were performed. In this case, the neurons were cultured with commercial EMEM medium plus 3 g/L of glucose under normal atmosphere (95% air/5% CO_2_). Thus, the controls were not under ODLG.

### 4.3. Reperfusion

After cells were subjected to ODLG for several periods of time (1, 3, 5, 12, 24 and 48 h), the medium was removed and replaced by normal medium (medium with serum plus antibiotics containing 3 g/L glucose plus 0.3 g/L glutamine). After this, cells were placed for 24 h, in a cell incubator containing 95% air/5% CO_2_ and 37 °C. At the end of this protocol, all parameters were evaluated for each experimental condition.

### 4.4. Assessment of Cell Viability

Cell viability was tested by crystal violet and the XTT assay.

#### 4.4.1. Crystal Violet Determination

Cortical neurons were washed twice with PBS (8.1 mM Na_2_HPO_4_, 2.7 mM KH_2_PO_4_, 173 mM NaCl, pH 7.4) and then exposed to 0.2% crystal violet in 2% ethanol for 20 min. After this time, cells were washed with distilled water until the excess dye was removed and then were lysed by adding 1% sodium dodecyl sulfate (SDS). Finally, absorbance was measured at 560 nm.

#### 4.4.2. XTT Determination

This assay is based on the ability of live metabolically active cells to reduce yellow tetrazolium salt (XTT) to form an orange formazan dye. Thus, this conversion can only occur in living cells. The newly-formed formazan dye is directly quantified using a scanning multi-well spectrophotometer at a wavelength of 492 nm (reference wavelength 690). The amount of orange formazan formed, as monitored by the absorbance, directly correlates to the number of living cells. Control and treated neurons were washed with PBS and incubated with the XTT solution (final concentration 0.3 mg/mL) according to Kit specifications. After this incubation period, orange dye solution was spectrophotometrically quantified at a wavelength of 492 nm (reference wavelength 690 nm). Results were expressed as percentages as compared to control cells, which were considered as 100%.

### 4.5. Measurement of ROS Formation

To assay ROS formation, 2,7-dichlorodihydrofluorescein diacetate (H_2_DCF-DA), a non-fluorescent lipophilic reagent, was used. H_2_DCF-DA enters the cells, where it is transformed into 2,7-dichlorodihydrofluorescein (H_2_DCF) by the action of intracellular stearases. H_2_DCF is oxidized to fluorescent DCF by hydrogen peroxide. H_2_DCF-DA (5 μM) was added to the cells, before subjecting to different ODLG conditions. After each treatment, the incubation medium was removed and the cells were washed twice with PBS. Finally, fluorescence was measured in an FL600-BioTek spectrofluorometer (Winooski, VT, USA) with filters of 485/20 nm excitation and 530/25 nm emission. Results are expressed as arbitrary fluorescence units (AFU).

### 4.6. Lactate Dehydrogenase (LDH) Release

For LDH determination, the culture media from the control and experimental cultures treatments were collected and the neurons lysed by adding 0.1 M Tris-HCl (pH 7.4), containing 0.1% Triton X-100, and then centrifuged at 13,000 rpm using an Eppendorf centrifuge. LDH activity was measured in culture medium as well as in the cells according to López *et al*. [52]. Activity of LDH release is given as a percentage compared to the total LDH content (LDH in the supernatant + LDH inside the cells).

### 4.7. Caspase-3 Activity Measurement

Control and treated cortical neurons were washed with PBS and lysed with cell lysis buffer (10 mM Tris-HCl, 10 mM NaH_2_PO_4_/Na_2_HPO_4_, pH 7.5, 130 mM NaCl, 0.5% Triton X-100, 10 mM Na_4_P_2_O_7_ and 2 mM dithiothreitol (DTT). Lysates were centrifuged at 13,000× *g* for 5 min. Caspase-3 activity was measured in the supernatants. Supernatants were incubated at 37 °C for 2–4 h in caspase-3 assay buffer (20 mM HEPES, pH 7.5, 10% glycerol, 2 mM DTT containing 20 μM Ac. *N*-acetyl-Asp-Glu-Val-Asp-(7-amino-4-methylcoumarin (AcDEVD-AMC). The fluorogenic 7-amino-methylcoumarin (AMC) liberated from Ac-DEVD-AMC was monitored using a spectrofluorometer Bio-Tek FL 600, at excitation wavelength of 360/20 nm and an emission wavelength range 460/20. Enzymatic activity is expressed as arbitrary fluorescence unit after 2 h per μg protein (AFU/2 h/μg protein). The amount of proteins in lysed cells was monitored by Bradford [53].

### 4.8. Cytochrome Oxidase Activity

After each treatment, the incubation medium was removed and cells were washed with PBS and lysed with 200 μL of 0.1 M potassium phosphate buffer pH 7.5 which contained 0.5% Triton X-100. The lysed cells were centrifuged at 13,000 rpm for 5 min before collecting supernatants. Reduced cytochrome c (7 mg) was dissolved in 1 mL of 0.1 M of potassium phosphate buffer pH 7.5 and dithionite was added to ensure cytochrome reduction. Excess dithionite was removed by passing the cytochrome c solution through a Sephadex G-25 column equilibrated with the buffer (Amersham Bioscience, Arlington, MA, USA). The eluyent was taken and adjusted to an optic density of 0.7 absorbance. The extract was added to 1 mL of this solution and absorbance measured at 550 nm. Activity was expressed as OD/10 min/mg protein.

### 4.9. Measurement of Glutathione

After treatments, the cultured medium was removed and cells washed with PBS. The cells were placed under ice and 300 μL of 0.1 M of sodium phosphate-EDTA pH = 8 and 100 μL of 25% phosphoric acid were added to each well. The cell suspension was centrifuged at 13,000× *g* for 5 min and the supernatant was taken. Glutathione concentration was expressed as AFU/μg protein. Reduced glutathione was evaluated following the protocol from Hissin and Hilf [54].

### 4.10. Measurement of Amino Acid Secretion

HPLC analysis of amino acids was performed following a procedure from Márquez *et al*. [55]. The cellular medium was removed and cells were washed twice with PBS. Cells were stimulated during 15 min at 37 °C with 0.5 mL of fresh Locke medium containing 60 mM KCl. After stimulation, the solution was taken and the amino acids in the solution were dansylated with dansyl chloride. Separation of dansyl-derivates was carried out in a 5 μM Spherisorb-ODS-2 column (15 × 0.46 cm; Sigma Aldrich, St. Louis, MO, USA) using a reversed-phase high-performance liquid chromatography with UV detection at 254 nm. Peaks were integrated using a Spectraphysic integrator (Sigma Aldrich) and quantified by comparison with simultaneously-prepared amino acid standards. Cells were lysed by adding 0.5 mL distilled water. The lysed cells were centrifuged at 13,000 rpm in an Eppendorf centrifuge (Hamburg, Germany) for 5 min and the supernatants were collected and proteins were measured. The amount of protein in the lysed cells was quantified using the Bradford assay [31]. Results were expressed as nmols of neurotransmitter/mg protein.

### 4.11. Membrane Potential Measurement

Changes in the membrane potential of neurons were monitored with the fluorescent dye bisoxonol (bis-[1,3-diethyl-thio-barbiturate]-trimethineoxonol), according to the Waggonet method [56]. The control neurons, and neurons after treatment were washed and incubated with 0.2 μM bisoxonol for 30 min. After this treatment, bisoxonol was removed and the cells were washed with PBS and suspended in PBS. Finally, fluorescence was measured at wavelengths of 540 nm excitation and 565 nm emission, and monitored with a FL600-BioTek spectrofluorimeter Fluorescence (Winooski, VT, USA) intensity was reported as arbitrary fluorescence units.

### 4.12. Mitochondrial Membrane Potential Assay

Mitochondrial membrane potential was measured following the protocol by Tenneti *et al*., [57], with minor modifications. Control and treated cells were washed with PBS and incubated for 30 min with 500 nM of tetramethylrhodamine methyl ester (TMRM) and dissolved in Locke medium (140 mM NaCl, 4.4 mM KCl, 2.5 mM CaCl_2_, 1.2 mM MgSO_4_, 1.2 mM KH2PO_4_, 4 mM NaHCO_3_, 5.5 mM glucose and 10 mM HEPES, adjusted to pH 7.5). Finally, cells were washed with PBS, and the fluorescence was measured using a FL600-BioTek spectrofluorimeter at 530/25 nm exc. and 590/20 nm em.

### 4.13. Analysis of Gene Expression

Total RNA was isolated from 5 × 10^6^ cells of primary neuron cultures, using a RNase mini kit from Qiagen, Hilden, Germany) following the manufacturer’s instructions with the optional RNase-free DNase step to avoid contamination with genomic DNA. RNA concentration was determined by spectrophotometer, reading absorbance at 260 nm and defining one optical density unit as equivalent to 40 μg/mL. The ratio between the absorbance values at 260 and 280 nm gave an estimation of RNA purity.

#### 4.13.1. RT-PCR Analysis

RNasy Mini Kit (Qiagen, Valencia, CA, USA) was used for total RNA isolation. Reverse transcription (RT) was carried out for 1 h at 55 °C with oligodeoxythymidylate primer using 5 μg of total RNA from each sample for complementary DNA synthesis.

Real time quantitative PCR to determine the levels of rat HIF1, HIF3, SOD1, GPX3 and housekeeping GAPDH mRNAs were performed using specific primers (See Table 1) synthesized at Sigma-Genosys (Oakville, ON, Canada).

#### 4.13.2. Real-Time PCR

The SYBR Green PCR Master Mix (Applied Biosystems, and the 7900 HT Fast Real-Time PCR System (Applied Biosystems, Foster City, CA, USA) were used to detect the real-time quantitative PCR products of reverse-transcribed cDNA samples, according to the manufacturer’s instructions. q-PCR conditions used are as follows: 95 °C (10 min), 40 cycles of 15 s at 95 °C and annealing for 1 min at 60 °C. Two independent quantitative PCR assays were performed for each gene and measured in triplicate. Two non-template controls were run for each quantitative PCR assay, and genomic DNA contamination of total RNA minus controls (samples without the reverse transcriptase).

### 4.14. Statistical Analysis

Data are presented as means ± SEM of three/four independent experiments using different cell cultures, each one performed in triplicate with different batches of cells. Statistical comparisons between two or more populations were made using the ANOVA on rank test to evaluate the normality and variance of the data. If both parameters are confirmed then the Holme-Sidak test detects significant differences between groups. When one of these parameters is absent, we did a Kruskal-Wallis non-parametric analysis as well as Dunn’s *pos Hoc* test using the SigmaPlot 11.0 software (Systat Software, Inc., San Jose, CA, USA).

### 4.15. Ethics Statement

Pregnant rats were obtained from the “Laboratory Animal form the Universidad Complutense of Madrid (U.C.M)”; License number #ES280790000086. The work was also approved by the University Animal Care Committee (C.E.A = Committee of Experimental Research and Ethics) from Universidad Complutense de Madrid (U.C.M); form number RD # 53/2013 for research and it was carried out in strict accordance with Guidelines for the Care and Use of Laboratory (European Council Directive 86/609/EEC). All surgery was performed under sodium pentobarbital anesthesia, and all efforts were made to minimize suffering of animals.

## 5. Conclusions

The major points of the investigation presented here may be stated as follows: (1) Cortical neurons subjected to ODLG conditions (1–5 h) do not die but remain in lethargic stage. This condition protects neurons by saving energy and nutrients; (2) During this ODLG time cortical neurons showed protective mechanisms in their upregulation of HIF-1α and HIF-3α production as well as of antioxidant enzymes (reduced glutathione, SOD-1 and GPX3), which could help cells recover during reperfusion; and (3) After long ODLG times (12–24 h), cortical neurons are under the same conditions as those at shorter ODLG times, but during this longer period there is cell damage and mitochondrial membrane alterations which do not allow recovery during reperfusion. Taken together, these molecular events in our ODLG model are in accordance with some molecular aspects reported within the penumbra of ischemic rats [58]. Consequently, drugs that can induce HIF-1 and SOD-1 could helpful to recover after short periods of ischemia.

## Figures and Tables

**Table 1.** mRNA primers.

mRNA	Primers
HIF1α	5′-GAAACTCCAAAGCCACTTCG-3′ (forward) 5′-CTGGCTGATCTTGAATCTGG-3′ (reverse)
HIF3α	5′-GCTTATCTGTGAAGCCATCC-3′ (forward) 5′-CAACTTCTGCAATCCTCTCG-3′ (reverse)
SOD1	5′-GATGAAGAGAGGCATGTTGG-3′ (forward) 5′-CCAATGATGGAATGCTCTCC-3′ (reverse)
GPX3	5′-CATGAAGATCCATGACATCC-3′ (forward) 5′-GATGTCCATCTTGACGTTGC-3′ (reverse)
GAPDH	5′-AAGGGCTCATGACCACAGTC-3′ (forward) 5′-TTCAGCTCTGGGATGACCTT-3′ (reverse)
